# Purkinje cell injury, structural plasticity and fusion in patients with Friedreich’s ataxia

**DOI:** 10.1186/s40478-016-0326-3

**Published:** 2016-05-23

**Authors:** Kevin C. Kemp, Amelia J. Cook, Juliana Redondo, Kathreena M. Kurian, Neil J. Scolding, Alastair Wilkins

**Affiliations:** Multiple Sclerosis and Stem Cell Group, Clinical Neurosciences office, University of Bristol, 1st floor, Learning and Research building, Southmead Hospital, Bristol, BS10 5NB UK; Brain Tumour research group, School of Clinical Sciences, University of Bristol, Bristol, BS10 5NB UK

**Keywords:** Friedreich’s ataxia, Purkinje cell, Cerebellum, Neurodegeneration, Fusion, Heterokaryon

## Abstract

Purkinje cell pathology is a common finding in a range of inherited and acquired cerebellar disorders, with the degree of Purkinje cell injury dependent on the underlying aetiology. Purkinje cells have an unparalleled resistance to insult and display unique regenerative capabilities within the central nervous system. Their response to cell injury is not typical of most neurons and likely represents both degenerative, compensatory and regenerative mechanisms. Here we present a pathological study showing novel and fundamental insights into Purkinje cell injury, remodelling and repair in Friedreich’s ataxia; the most common inherited ataxia. Analysing post-mortem cerebellum tissue from patients who had Friedreich's ataxia, we provide evidence of significant injury to the Purkinje cell axonal compartment with relative preservation of both the perikaryon and its extensive dendritic arborisation. Axonal remodelling of Purkinje cells was clearly elevated in the disease. For the first time in a genetic condition, we have also shown a disease-related increase in the frequency of Purkinje cell fusion and heterokaryon formation in Friedreich's ataxia cases; with evidence that underlying levels of cerebellar inflammation influence heterokaryon formation. Our results together further demonstrate the Purkinje cell’s unique plasticity and regenerative potential. Elucidating the biological mechanisms behind these phenomena could have significant clinical implications for manipulating neuronal repair in response to neurological injury.

## Introduction

Purkinje cells represent the sole output neuron of the cerebellar cortex and thus changes in their function have significant impact on the function of the cerebellum as a whole. Purkinje cell pathology is a common finding in a range of inherited and acquired cerebellar disorders, with the degree of Purkinje cell injury dependent on the underlying aetiology. The inherited ataxias are a group of genetically heterogeneous conditions that share common clinical features that give rise to progressive ataxia, with cerebellar injury and varying degrees of degeneration of other grey matter regions. Despite advances in detection of precise genetic causes, no therapy has been shown to prevent or slow the progression of disability in these disorders.

Friedreich ataxia (FRDA) is the commonest autosomal recessive ataxic condition, with 95 % of cases caused by a homozygous GAA.TCC tri-nucleotide repeat expansion mutation within intron 1 of the *FXN* gene [6] leading to transcriptional repression of the mitochondrial protein frataxin [15, 46]. Patients with FRDA experience insidious accumulation of neurological disability with progressive trunk and limb ataxia, dysarthria, sensory neuropathy and pyramidal weakness [17]. Neuropathologically, prominent areas of degeneration associated with the disease are the dorsal root ganglia, peripheral nerves, spinal cord, and cerebellum [23]. Hypoxic-ischemic damage, due to cardiomyopathy or pulmonary complications, may also result in secondary brain injury. The most significant lesion of the central nervous system (CNS) is found within the dentate nucleus, located within the deep white matter of each cerebellar hemisphere. Selective atrophy of the large neurons and their efferent myelinated fibres within the dentate nucleus is severe, and is accompanied by abnormal dendritic expansion and proliferation of the corticonuclear gamma-aminobutyric acid (GABA)-ergic terminals about the dendrites of dying neurons, termed ‘grumose degeneration’. Remarkably, neuronal loss within the dentate nucleus does not result in a significant level of retrograde atrophy within the Purkinje cell population and the cerebellar cortex is generally intact [27]. Nevertheless, in some patients, Purkinje cell arborisation defects have been reported and mild loss of these cells can be seen at end-stage disease [25, 39].

Purkinje cells have a fairly unique and unparalleled resistance to axonal injury within the CNS [12]. Their response to insult is not typical of most neurons and likely represents both degenerative, compensatory and regenerative mechanisms. Pathological aberrations to Purkinje cell morphology have been observed in cerebellar disease, including axon torpedo formation and loss in cyto-architecture [24, 33, 35, 44]. Structural plasticity in the form of axon remodelling and intra-cortical branching can occur in Purkinje cells and axonal sprouting to establish contact with surviving cells has been reported in humans [1], which may represent a potential mechanism by which cells attempt to re-establish cellular connections and access trophic support [43]. The phenomenon of bone marrow-derived cells (BMDCs) fusing with Purkinje cells to form bi-nucleate heterokaryons has also been observed in a variety of experimental models of cerebellar disease [2, 3, 8, 10, 11] and also in patients with multiple sclerosis [22]. Accumulating evidence is raising new questions into the biological significance of cell fusion, with the possibility that it represents an important physiological phenomenon to rescue damaged neurons [36, 51].

Understanding whether Purkinje cell axon remodelling and/or fusion represent mechanisms by which cerebellar functions can be maintained in genetic cerebellar disease has important therapeutic consequences. With the potential to protect and rescue neuronal cells and restore homeostatic balance during neurodegeneration, understanding the circumstances in which they occur may lead to techniques to manipulate these mechanisms therapeutically. With this in mind, using post-mortem cerebellum tissue, our aims were to quantify the extent of Purkinje cell injury and structural plasticity in FRDA, a condition typically associated with Purkinje cell preservation, in order to explore whether plasticity and fusion might contribute to Purkinje cell survival.

## Materials and methods

### Patients

**Fig. 1:**
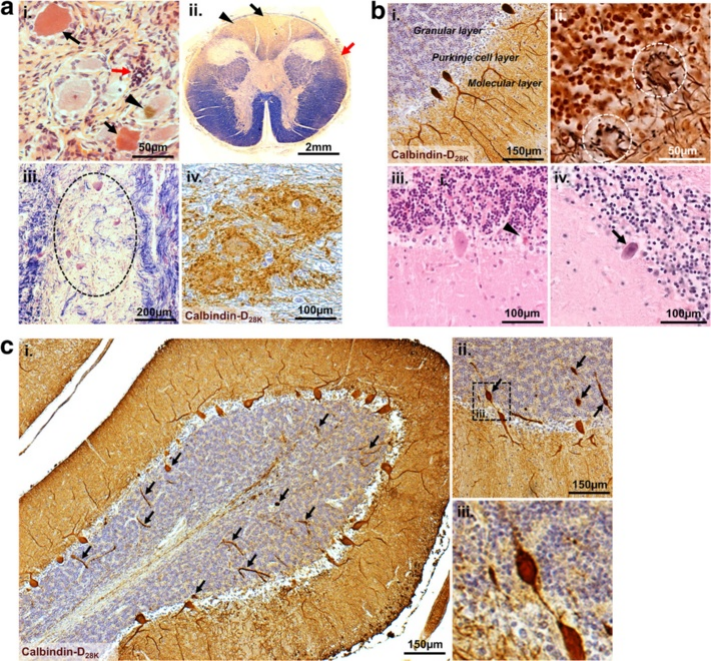
Neuronal injury in FRDA. Tissue sections derived from FRDA cases showing pathological lesions. **a** Characteristic FRDA lesions. **(i)** H&E staining of the dorsal root ganglion showing shrunken neurones with severe eosinophilia of the perikaryon (black arrows), neuronal lipofuscin accumulation (black triangle) and a nest of reactive satellite cells known as a nodule of Nageotte indicating ganglion neuronal cell degeneration (red arrow). **(ii)** A section from the upper cord stained with Luxol fast blue and Cresyl violet showing marked dorsal column loss, more marked in the gracile (black arrow) than the cuneate fasiculi (black triangle). There is also significant degeneration of the posterior spinocerebellar tracts (red arrow). **(iii)** Luxol fast blue and Cresyl violet staining of the spinal cord showing loss of neurons from Clarke’s Column (black hatched area). **(iv)** Grumose degeneration; disorganisation and proliferation of the Calbindin-D_28K_ positive synaptic terminals situated around the large neurons of the cerebellar dentate nucleus. **b** Signs of Purkinje cell injury. **(i)** Cerebellar sections immuno-labelled with Calbindin-D_28K_ and counterstained with haematoxylin (blue) showing the location of the Purkinje cell layer situated within the grey matter on the border of the molecular and granular cell layers. (**ii**) Bielschowsky silver staining of the cerebellar cortex showing occasional loss of Purkinje cells leading to the presence of ‘empty baskets’ (white hatched areas). H&E staining showing the Purkinje cell layer with (**iii**) occasional shrunken Purkinje neurones with mild eosinophilia of the perikaryon (black triangle), and (**iv**) Purkinje cells with loss of Nissl substance and shrunken basophilic homogenous nuclei (black arrow). **c** (**i**) A cerebellar folium showing frequent Calbindin-D_28K_ positive hypertrophic axons situated throughout the grey matter (black arrows). (**ii**) Cerebellar cortex showing Calbindin-D_28K_ positive axonal torpedoes (black arrows). The hatched area in (**ii**) represents the higher magnified image (**iii**)

Post-mortem cerebellum samples from eight patients with FRDA and five control patients were obtained through collaboration with both *BRAIN UK* at the University of Southampton, Southampton, UK and *The UK Multiple Sclerosis Tissue Bank* at the Imperial College, London, UK. The majority of cases pre-dated genetic testing for FRDA and information regarding the GAA.TCC tri-nucleotide repeat expansion lengths for each FRDA case were not available. As a result, patients had been clinically diagnosed as having FRDA and diagnosis had been confirmed during neuropathological autopsy examination. Neuropathological reports at post-mortem included: axonal loss and prominent gliosis in the dorsal columns (more marked in the gracile than the cuneate fasciculus), long tracts and spinocerebellar tracts of the spinal cord; neuronal loss in the dorsal nucleus of Clarke; gliosis within the white matter of the cerebellum; neuronal shrinkage and loss in the dentate nucleus with consequent atrophy of the superior cerebellar peduncles; and marked depletion and gliosis of the gracile and cuneate nuclei, vestibular nuclei and solitary tract of the medulla. In the periphery, neuronal loss in the dorsal root ganglia was documented and peripheral nerves also showed demyelination and axonal degeneration. For several FRDA cases, stained spinal cord and DRG post mortem sections were available. Thus where possible, representative images of highly specific FRDA lesions in the spinal cord, DRG were taken (Fig. 1a). Control cerebellum samples were derived from patients who had died from causes other than neurological disease (Table 1). All tissues were collected with the donors’ fully informed consent via a prospective donor scheme. At death, brains were removed, fixed in neutral buffered formalin and tissue blocks embedded in paraffin. For this study, 10 μm sections were cut from cerebellar tissue of each patient and mounted onto glass slides. In 10 μm sections, approximately half of a Purkinje cell perikaryon could be encompassed.

**Table 1:** The characteristics of both FRDA and control cases used within this study

Case	Pathological diagnosis	Sex (M/F)	Age (years)	Cause of death
1	FRDA	F	33	Heart Failure
2	FRDA	M	46	Pneumonia
3	FRDA	M	50	Pneumonia
4	FRDA/FRDA variant	F	66	Pneumonia
5	FRDA	F	42	Pneumonia
6	FRDA	M	51	Pulmonary Embolism
7	FRDA	M	47	Not known
8	FRDA	F	n/a	Not known
*mean*			47.9	
*SE*			4.5	
1	Control	M	82	Not known
2	Control	M	88	Prostate cancer, bone metastases
3	Control	M	68	Heart failure, fibrosing alveolitis, coronary artery artheroma
4	Control	M	84	Bladder cancer, pneumonia
5	Control	M	82	Myelodysplastic syndrome, rheumatoid arthritis
*mean*			80.8	
*SE*			3.4	

### Histochemical stains

For histological assessment, sections were deparaffinised in Clearene, dehydrated in 100 % ethanol, hydrated in distilled water, and stained with haematoxylin and eosin (H&E), Bielschowsky silver staining or Luxol fast blue with Cresyl violet.

### Immunohistochemistry

Cerebellar sections were immuno-stained with antibodies to rabbit anti-Calbindin-D_28K_ (1:500) (Sigma-Aldrich, UK) and HLA-DP DQ DR (1:800) (Dako, Cambridgeshire, UK). Sections were deparaffinised in Clearene, dehydrated in 100 % ethanol, hydrated in distilled water, and immersed in 3 % hydrogen peroxide in methanol for 30 min to block endogenous peroxidase activity, rinsed and microwaved in sodium citrate buffer (0.01 M, pH 6.0, 5 min) or EDTA buffer (1 mM, pH 8, 10 min) as appropriate and rinsed in phosphate-buffered saline (PBS). Non-specific binding was blocked with Vectastain blocking serum (20 min). After addition of the primary antibody, sections were incubated overnight at 4 °C. The sections were then rinsed in PBS before incubation for 20 min with secondary antibody (Vectastain Biotinylated Universal antibody) and 20 min with VectaElite ABC Complex (PK-6200, Vector Laboratories, Peterborough, UK) followed by a 10-min incubation with 3,3′-diaminobenzidine (DAB) and 0.01 % H_2_O_2_. Sections were washed in water, immersed in copper sulphate DAB enhancer (4 min), counterstained with haematoxylin, dehydrated, cleared and mounted. Images were acquired using an Olympus IX70 microscope coupled with Image-Pro Plus software.

### Immunofluorescence labelling

Sections were deparaffinised, hydrated and washed as above. An antigen retrieval step was performed through microwaving in sodium citrate buffer (0.01 M, pH 6.0, 5 min). Purkinje cells were labelled by single or double immunofluorescence using rabbit anti-Calbindin-D_28K_ (1:500) (Sigma-Aldrich, UK), mouse anti-SMI-34 (phosphorylated neurofilament; 1:500) (Covance, US) and rat anti-myelin basic protein (MBP) (1:100) (Serotec, UK). Non-specific binding was blocked with 10 % normal goat serum diluted in PBS containing 0.1 % triton. Sections were incubated at 4 °C overnight with primary antibodies. Sections were then washed in PBS and incubated for 30 min in the dark with Alexa Fluor 555, goat anti-mouse (1:500), Alexa Fluor 488, goat anti-rabbit (1:500) or Alexa Fluor 555, goat anti-rat (1:500) (Invitrogen, Paisley, UK), before being washed in PBS and mounted in Vectashield medium containing the nuclear dye 4′6′-diamidino-2-phenylindole (DAPI) (H-1200, Vector Laboratories). Sections were imaged using either: 1) a Leica SP5-AOBS confocal laser scanning microscope attached to a Leica DM I6000 inverted epifluorescence microscope with Leica Application Suite Advanced Fluorescence software and Volocity 3D image software (PerkinElmer, USA); or 2) a Nikon C1 confocal microscope and EZ viewer software.

### Microscopy

#### Quantification of Purkinje cells

The total number of Purkinje cells was counted in FRDA and control sections, scanning the entire length of the Purkinje cell layer. Purkinje cells were identified based on Calbindin positivity and their specific location. To account for the difference in section sizes and plane of cutting, the length of the Purkinje cell layer was traced and measured in each section using Image J software to obtain the number of Purkinje cells per standardized unit length (mm). For these sections, the length of Purkinje cell layer was measured and the number of Purkinje cells in each area counted.

#### Quantification of SMI-34 positive Purkinje cell perikarya and axonal spheroids

The total number of axonal spheroids and Purkinje cell perikarya positive for SMI-34 were counted in FRDA and control sections, scanning the entire Purkinje cell and granular layers.

#### Purkinje cell morphology

Using Image J software, the outline of each Purkinje cell perikaryon (where the nucleus was clearly visible) was traced and the area within subsequently calculated. The total number of Purkinje cells with an abnormal perikaryal morphology were also counted in FRDA and control sections, scanning the entire length of the Purkinje cell layer. To investigate changes in dendritic arborisation, concentric circles (with increasing diameter of 20 μm) were aligned on the centre of each Purkinje cell perikaryon and the number of times each circle transected a dendrite was counted. Subsequently, the number of transections per mm at each distance was calculated. As the dendritic tree of Purkinje cells is not spherical, only section orientations that resulted in a full display of Purkinje cell arbor to be visualised were used for quantification.

#### Purkinje cell axonal morphology

Specific changes in Purkinje cell axonal morphology were counted in both FRDA and control sections, scanning the entire length of the Purkinje cell and granular layers. Changes included the number of Calbindin-D_28K_ positive thickened axons (a minimum 2-fold increase in calibre of normal appearing axons), thickened recurrent collaterals (hypertrophic collateral branch attached to the corticofugal axon with a minimum 90° turn back towards the Purkinje cell layer) and thickened arciform fibres (hypertrophic axons with a minimum 90° turn back towards the Purkinje cell layer). Both Calbindin-D_28K_ positive axonal branching (axons with at least one branch point not defined as a collateral) and terminal axonal sprouting (a network of fine nodular/branched processes projecting from the intact axon) were also counted [1, 43]. For these sections, the length of Purkinje cell layer was measured and the number of Purkinje cells in each area calculated. All axonal changes were then expressed as either frequency per standardized unit length (100 μm) or frequency per Purkinje cell.

#### Quantification of Purkinje cell heterokaryons

For identification of bi-nucleate Purkinje cell heterokaryons, cerebellar sections immuno-labelled for Calbindin-D_28K_ were viewed using a Leica SP5-AOBS confocal laser scanning microscope attached to a Leica DM I6000 inverted epifluorescence microscope. Each section was scanned along the entire length of the Purkinje cell layer, situated between the granular cell layer and molecular layer, for Purkinje cell bodies containing two separate nuclei. At least 2000 Purkinje cells from each patient sample were examined, allowing for the determination of the frequency of bi-nucleate Purkinje cells. All bi-nucleate Purkinje cells were confirmed on the confocal microscope by obtaining serial sections throughout the whole Purkinje cell body. All Z-stack and 3-dimensional imaging was created using both Leica Application Suite Advanced Fluorescence software and Volocity 3D image software (PerkinElmer, USA).

#### Quantification of microglial cells

All HLA-DP DQ DR positive cells were counted, using at least 18 randomly assigned fields per case, within the white matter and grey matter regions of the cerebellum. The number of HLA-DP DQ DR positive cells per mm^2^ was subsequently calculated.

### Statistical analysis

The analysis was performed using GraphPad Prism (GraphPad Software Inc, USA). For all tests, values are expressed as the mean ± SE from at least five independent cases, taking *P* <0.05 to represent statistical significance. Data between two groups were analysed using either unpaired t-tests or Mann-Whitney U tests. Statistical comparisons for over two groups were analysed using either one-way or two-way analysis of variance (ANOVA) with *post hoc* testing between groups where appropriate. Pearson’s correlation was used to analyse relationships between two sets of data.

## Results

### Identification of cerebellar Purkinje cells

To identify Purkinje cells within cerebellar tissue, sections were immuno-labelled for the calcium-binding protein Calbindin-D_28K_. Purkinje cells were readily identified based on their unique morphology and location; their perikaryon situated within the Purkinje cell layer positioned between the granular cell and molecular layers (Fig. 1b). In control cases, Purkinje cells showed a typical morphology; with extensive dendritic arborisation climbing through the molecular layer toward the surface of the cortex, with its axon originating at the opposite (basal) pole of the cell perikaryon, passing through the granular layer (where it becomes myelinated) towards the white matter, sending inhibitory efferent projections to the deep cerebellar nuclei.

### Purkinje cell injury in Friedreich’s ataxia

In FRDA cases, Purkinje cells showed several distinct features. Disorganisation and proliferation of Calbindin-D_28K_ positive synaptic terminals surrounding soma and dendrites of the dentate neurons, termed ‘grumose degeneration’ was present (Fig. 1a). Using H&E staining, the Purkinje cell layer showed occasional shrunken Purkinje neurones with mild eosinophilia of the perikaryon, loss of Nissl substance and shrunken basophilic homogenous nuclei (Fig. 1b). The occasional loss of Purkinje cells leading to the presence of ‘empty baskets’ (the axon terminals of basket cells that typically enwrap the Purkinje cell perikaryon appear to surround an empty space) were observed using Bielschowsky silver staining (Fig. 1b). A marked incidence of Purkinje cell axonal thickening and swellings (torpedoes) situated within the granular layer was also evident (Fig. 1c).

**Fig. 2:**
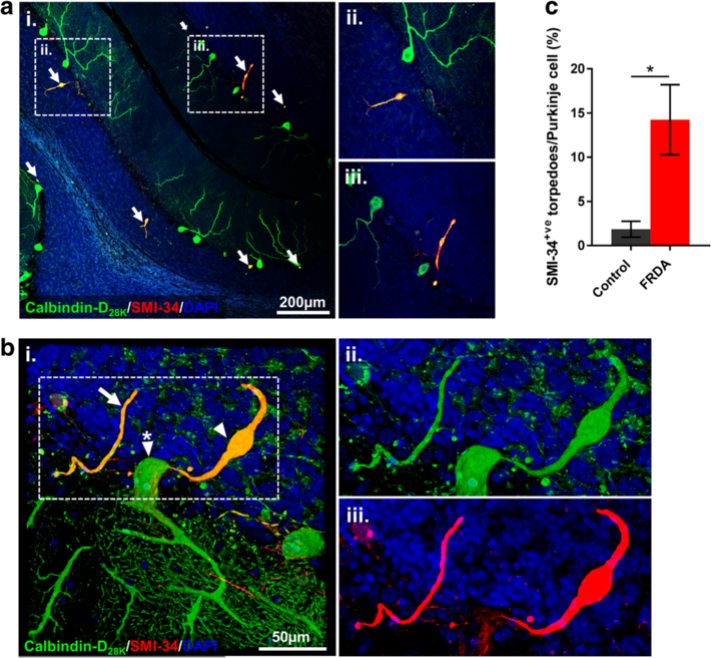
Abnormal neurofilament phosphorylation within Purkinje cell axons in FRDA. **a** A representative image of the cerebellar folia from an FRDA case showing Calbindin-D_28K_ positive hypertrophic axons and axonal torpedoes expressing high levels of SMI-34. The hatched areas in (**i**) represents the higher magnified images (**ii**/**iii**). White arrows in (**i**) indicate areas of Calbindin-D_28K_/SMI-34 co-expression. **b** 3-dimensional confocal image of a Purkinje cell immunofluorescently labelled with Calbindin-D_28K_ (green), SMI-34 (red) and DAPI nuclear stain (blue) (perikaryon (white triangle/asterisk), axonal torpedo (white triangle), recurrent collateral (white arrow)). The hatched areas in (**i**) represents the higher magnified images (**ii/iii**). **c** Quantification of SMI-34 positive torpedoes per Purkinje cell in FRDA and control cases. Results are expressed as the mean +/- (SE) (*p < 0.05, comparing FRDA to control)

Dual-immunolabelling of cerebellar sections with Calbindin-D_28K_ and SMI-34 (anti-phosphorylated neurofilament) was used to further characterise Purkinje cell axonal morphology and injury [44]. FRDA cases had noticeable changes in axonal morphology (hypertrophy or swelling of the axon segment) compared to controls; these structures appearing to arise from the proximal end of the corticofugal axon, being strongly immuno-reactive for both Calbindin-D_28K_ and SMI-34 (Fig. 2a and b). A small number of Calbindin-D_28K_/SMI-34 torpedoes were seen in control cases, however the number of Calbindin-D_28K_/SMI-34 torpedoes per Purkinje cell body was significantly increased in FRDA cases compared to controls (*p* <0.05) (Fig. 2c).

**Fig. 3:**
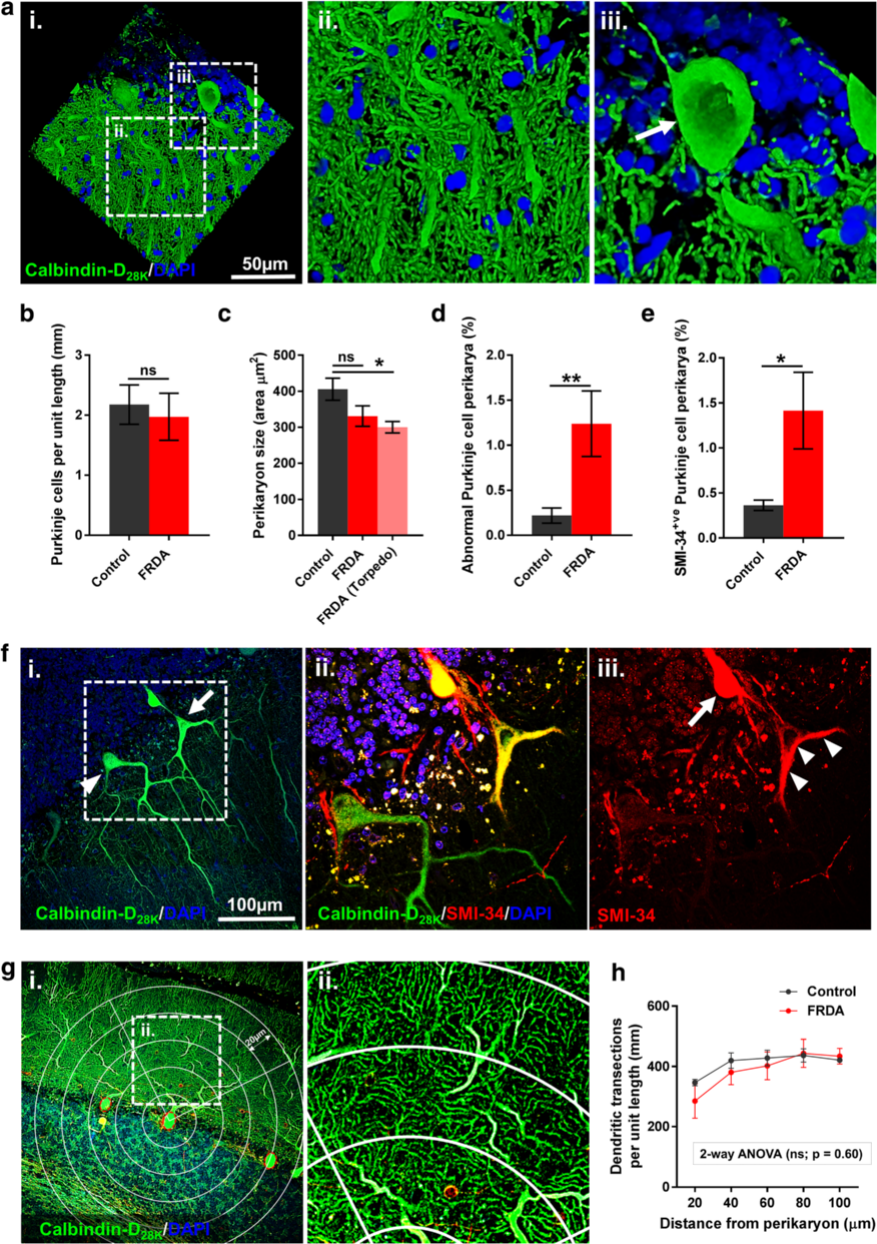
Analysis of both the Purkinje cell perikaryal and dendritic compartments in FRDA. **a** 3-dimensional confocal image of a FRDA case depicting a Purkinje cell immunofluorescently labelled with Calbindin-D_28K_ (green) and DAPI nuclear stain (blue). The hatched areas in (**i**) represents the higher magnified images (**ii**; dendritic compartment) and (**iii**; perikaryal compartment (white arrow)). Quantification of (**b**) Purkinje cells per mm in FRDA and control cases; (**c**) Purkinje cell perikaryon cell size categorised into: total Purkinje cells in FRDA (FRDA), Purkinje cells in FRDA with an axon torpedo (FRDA torpedo) and total Purkinje cells in control cases; (**d**) Purkinje cells with an abnormal perikaryal morphology; and (**e**) Purkinje cells with SMI-34 positive perikarya. **f**, **(i)** Purkinje cell with a normal perikaryon morphology (white triangle) and a Purkinje cell with an axon torpedo presenting with an abnormal (deflated and shrunken appearance) perikaryal morphology (white arrow). The hatched areas in (**i**) represents the higher magnified images (**ii**) and (**iii**) showing the shrunken Purkinje cell with axonal (white arrow) and perikaryal/dendritic SMI-34 accumulation (white triangles). **g** A representative image depicting how changes in dendritic arborisation were investigated. Concentric quarter circles (with increasing diameter of 20 μm) were aligned on the centre of each Purkinje cell perikaryon and the number of times each circle transected a dendrite was counted. The number of transections per mm at each distance was calculated. The hatched areas in (**i**) represents the higher magnified image (**ii**). **h** Quantification of the number of dendritic transections per mm at increasing distances from the cell perikaryon in FRDA and control cases. Results are expressed as the mean +/- (SE) (**p < 0.01, *p < 0.05, ns (not significant), comparing FRDA to control)

Morphological changes in both the Purkinje cell perikaryon and dendritic compartments were examined (Fig. 3a-h). No significant differences were found in the number of Purkinje cells per unit length (mm) of the Purkinje cell layer (Fig. 3b). In addition, no differences in overall Purkinje cell perikaryon size were evident between patient groups (Fig. 3c). However, Purkinje cells in FRDA cases with an associated axon torpedo showed a significant reduction in perikaryon size when compared to controls (*p* <0.05) (Fig. 3c). This sub-group of Purkinje cells commonly displayed a ‘deflated’ and shrunken perikaryal morphology (Fig. 3f); a proportion of these cells also presented with marked SMI-34 accumulation within their perikaryon/dendrites (Fig. 3f). Both the percentage of Purkinje cells with abnormal perikaryal morphology (Fig. 3d) and the percentage of Purkinje cells with SMI-34 positive perikarya (Fig. 3e) were significantly elevated in FRDA cases compared to controls (*p* < 0.05). Dendritic arborisation of Purkinje cells is a critical determinant of neuronal connectivity and health [7, 34]. To investigate changes in dendritic arborisation, the number of dendrites at increasing distances (20-μm increments) from the Purkinje cell perikaryon was counted (Fig. 3g). Again no differences in dendritic transections were found in FRDA cases compared to controls (*p* <0.05) (Fig. 3h).

### Evidence of Purkinje cell structural remodelling and myelin disruption in FRDA

**Fig. 4:**
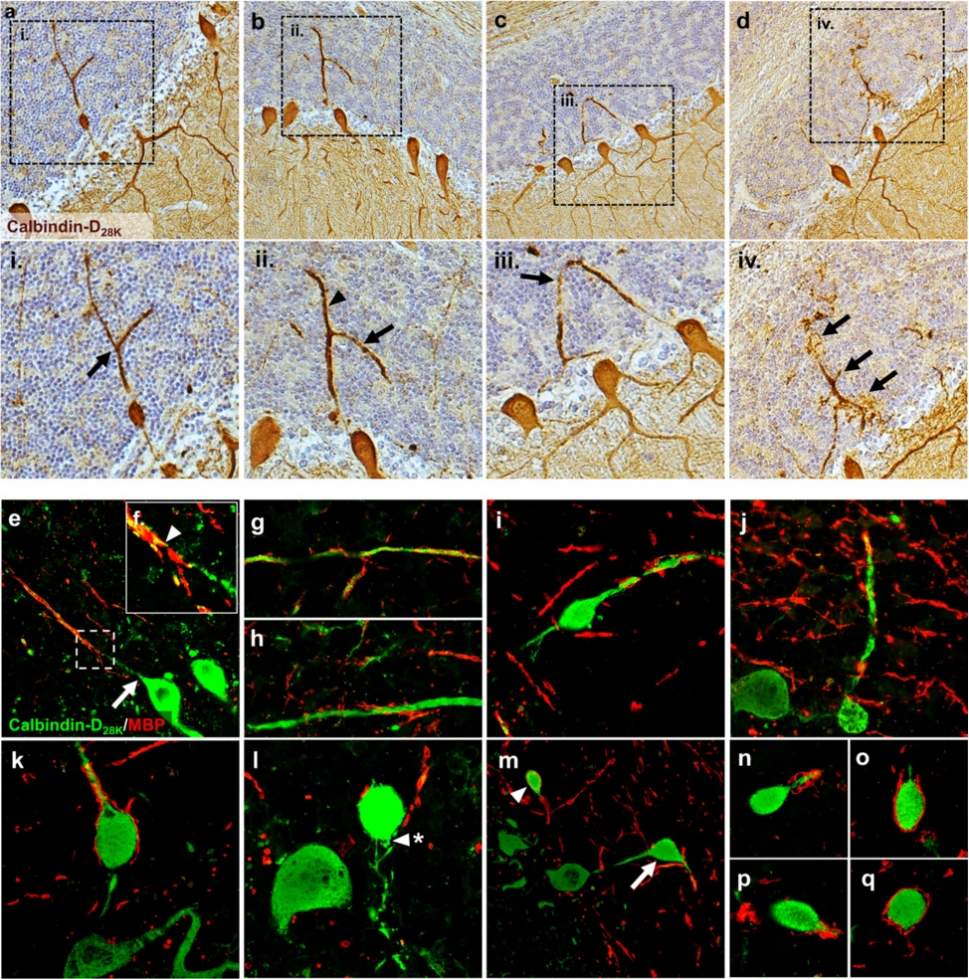
Purkinje cells in FRDA show indications of both axonal remodelling and myelin disruption. Unless stated, all cerebellar images are from FRDA cases. **a**-**d** Sections are DAB (brown) immuno-labelled with Calbindin-D_28K_ and counterstained with haematoxylin (blue). **e-q** Sections are immunofluorescently labelled with Calbindin-D_28K_ (green) and MBP (red). The hatched areas in **a**, **b**, **c**, **d** represents the higher magnified images (**i**), (**ii**), (**iii**), (**iv**) respectively. **a** Axonal thickening and branching (**i**; black arrow). **b** Thickening of both axon (**ii**; black triangle) and recurrent collateral (**ii**; black arrow). **c** Hypertrophic axon with arciform profile (**iii**; black arrow). **d** Axonal branching with terminal sprouting (**iv**; black arrows). **e** A typically ‘normal’ Purkinje cell axon, from a control case, projecting from the perikaryon (white arrow), through the granular layer where it become myelinated (white triangle). The hatched area represents the higher magnified image **f**. Abnormal hypertrophic axons showing either **g** fragmented myelin along the axon or h complete myelin loss. **i**-**k** Purkinje cell axons showing both axonal hypertrophy and fragmented myelin distal to axon torpedoes. **l** A Purkinje cell torpedo absent of myelin and showing indications of sprouting (white triangle/asterisk). **m** Myelinated (white triangle) and non-myelinated (white arrow) Purkinje cells torpedoes. **n**-**q** Purkinje cell torpedoes with varying degrees of myelin fragmentation

Atypical compensatory responses of Purkinje cells to axonal injury may occur in response to cerebellar injury. In control and FRDA cases, structural remodelling of Purkinje cell axons was observed with evidence of hypertrophic axons, hypertrophic recurrent collaterals and arciform fibres (Fig. 4a, b and c). Both axonal branching and axonal sprouting were also visible in both Purkinje cells with and without axonal torpedoes (Fig. 4a and d). These structural changes were significantly elevated, when normalised to the Purkinje cell layer length and/or Purkinje cell number, in FRDA cases compared to controls (*p* <0.05) (Table 2).

**Table 2:** Structural remodelling of Purkinje cell axons in both FRDA and control cases

Axonal change	Comparator	Control cases (Mean +/-SE (median))	FRDA cases (Mean +/-SE (median))	Significance (*p* value)
Hypertrophic axons	*Length*	0.279 +/- 0.216 (0.298)	1.642 +/- 0.659 (1.028)	*p* < 0.010
	*Purkinje cells*	0.016 +/- 0.007 (0.010)	0.054 +/- 0.018 (0.036)	*p* < 0.050
Hypertrophic recurrent collaterals	*Length*	0.115 +/- 0.038 (0.118)	0.457 +/- 0.143 (0.396)	*p* < 0.050
	*Purkinje cells*	0.003 +/- 0.001 (0.003)	0.015 +/- 0.004 (0.011)	*p* < 0.050
Arciform fibres	*Length*	0.029 +/- 0.014 (0.023)	0.158 +/- 0.052 (0.138)	*p* < 0.050
	*Purkinje cells*	0.001 +/- 0.001 (0.001)	0.005 +/- 0.001 (0.004)	*p* < 0.050
Axonal branching	*Length*	0.064 +/- 0.024 (0.060)	0.271 +/- 0.088 (0.261)	*p* = 0.082
	*Purkinje cells*	0.002 +/- 0.001 (0.002)	0.009 +/- 0.003 (0.006)	*p* < 0.050
Axonal sprouting	*Length*	0.062 +/- 0.027 (0.060)	0.226 +/- 0.078 (0.172)	*p* = 0.052
	*Purkinje cells*	0.002 +/- 0.001 (0.002)	0.006 +/- 0.001 (0.007)	*p* < 0.050

The frequency of axonal structural changes within the granular and Purkinje cell layers of both control and FRDA cases. All axonal changes are expressed as either frequency per standardized unit length of the Purkinje cell layer (100 μm) (*Length*) or frequency per Purkinje cell (*Purkinje cells*). Comparisons between control and FRDA were analysed using Mann-Whitney U tests

Using MBP and Calbindin-D_28k_ co-labelling, we also observed significant changes in Purkinje cell axonal myelination within the granular layer, with evidence of myelin fragmentation and loss localised to areas of axonal hypertrophy and axonal torpedoes (Fig. 4e-q).

### Quantification of bi-nucleate Purkinje cell heterokaryons

**Fig. 5:**
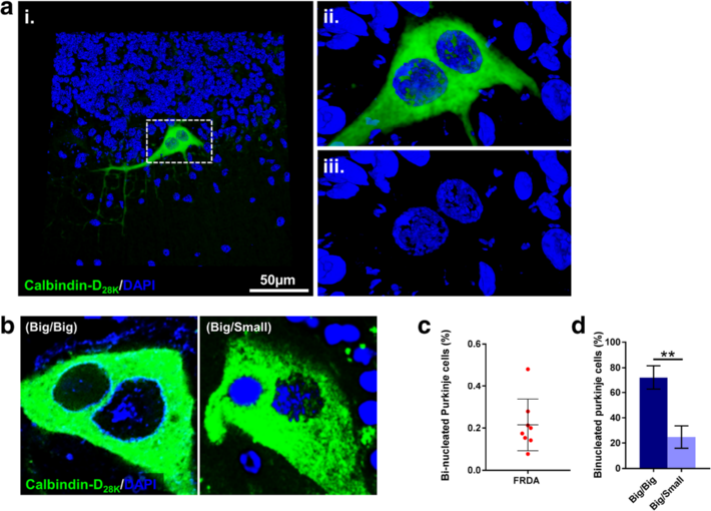
An increase in bi-nucleate Purkinje cells is evident within the cerebellum of FRDA cases. **a** 3-dimensional confocal image of a bi-nucleate Purkinje cell immunofluorescently labelled with Calbindin-D_28K_ (green) and DAPI nuclear stain (blue). The hatched area in (**i**) represents the higher magnified images (**ii**/**iii**). **b** A representative image of a bi-nucleate Purkinje cell containing two large Purkinje cell-like nuclei (Big/Big) and a bi-nucleate Purkinje cell with one large Purkinje cell-like nucleus and one smaller nucleus with compact chromatin (Big/Small). **c** Quantification of bi-nucleate Purkinje cells in FRDA cases. **d** Quantification of bi-nucleate Purkinje cells in FRDA cases categorised by their nuclear phenotype (Big/Big) or (Big/Small). Results are expressed as the mean +/- (SE) (**p < 0.01, comparing FRDA to control or Big/Big to Big/Small)

Using Calbindin-D_28k_ labelling and DAPI nuclear stain, sections from FRDA cases were scanned along the entire length of the Purkinje cell layer for Purkinje cells containing two nuclei (bi-nucleate heterokaryons). Using epifluorescence microscopy, we examined ≥2000 Purkinje cells from each case (total = 22,829 Purkinje cells). The location of each suspected bi-nucleate Purkinje cell was noted and subsequently scanned using confocal microscopy acquiring 0.20 μm serial sections throughout the entire Purkinje cell soma (Fig. 5). Following confocal evaluation and serial reconstruction, bi-nucleate Purkinje cells were found to be present in all FRDA cases. The frequency of bi-nucleate Purkinje cells detected in patients with FRDA was low (mean 0.215 % +/- 0.043) (Fig. 5c). Comparing this to previously published data reported by our research group, describing the frequency of bi-nucleate Purkinje cells detected in the same control patient cohort (mean 0.024 % +/- 0.006) [22], we report a significant increase in the incidence of bi-nucleate Purkinje cells in FRDA when compared to control cases (*p* <0.01) (Fig. 5c).

As previously reported [54], two distinct types of nuclear morphology were observed in bi-nucleate Purkinje cells; either a large Purkinje cell-like nucleus with disperse chromatin and a large nucleolus or a smaller nucleus (a little more than half the normal size) with compact chromatin (Fig. 5b). In patients with FRDA the majority (70.21 % +/-9.40) of bi-nucleate cells contained two large Purkinje cell-like nuclei (*p* <0.05). All other bi-nucleate Purkinje cells contained one large Purkinje cell-like nucleus and a second smaller nucleus with compact chromatin (Fig. 5b and d). Using correlative analysis, in FRDA cases we found no significant correlations between the frequency of bi-nucleate Purkinje cells with either patient age at death or the number of Calbindin-D_28K_/SMI-34 torpedoes per Purkinje cell body (data not shown).

### Cerebellar inflammation in FRDA

**Fig. 6:**
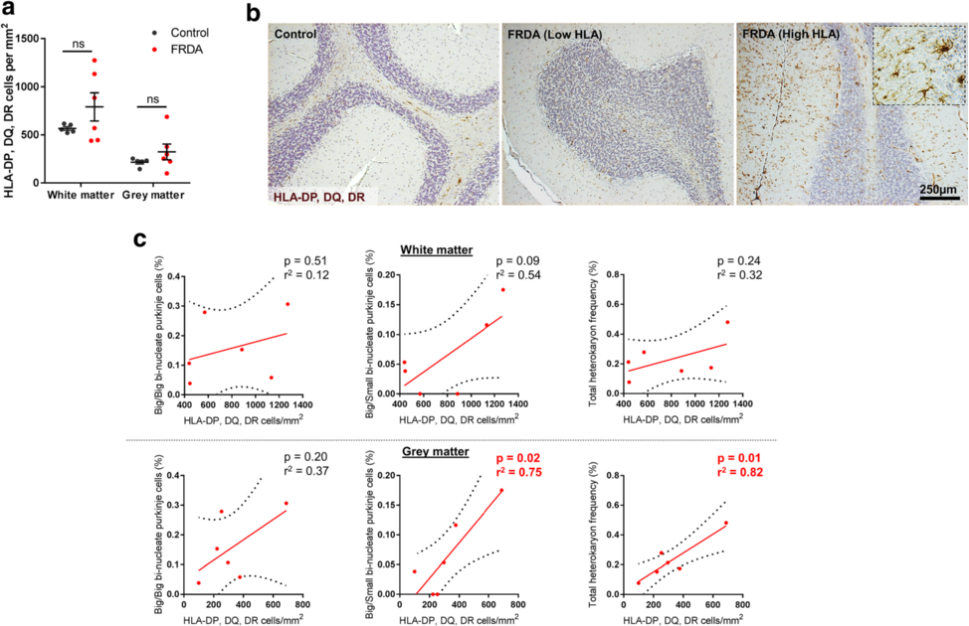
Microglial numbers within cerebellar grey matter regions positively correlate with the incidence of bi-nucleate Purkinje cells. **a** Levels of HLA-DP, DQ, DR positive cells within the cerebellar white and grey matter regions of FRDA and control cases. **b** Representative images of HLA-DP, DQ, DR positive cells (DAB (brown) immuno-labelled and counterstained with haematoxylin (blue)) within the cerebellar folia of FRDA and control cases. The dashed inset is a higher magnified image depicting microglial morphology in a FRDA case showing microglia with both enlarged perikarya and thicker processes. **c** Correlations and linear regression analysis between microglial cell numbers (in both the white matter and grey matter) and the frequency of total bi-nucleate Purkinje cells, bi-nucleate Purkinje cells with two large Purkinje cell-like nuclei (Big/Big) or bi-nucleate Purkinje cells with one large Purkinje cell-like nucleus and one smaller nucleus with compact chromatin (Big/Small). Results are expressed as the mean +/- (SE). *R* ^2^ = Pearson’s correlation coefficient of determination. (ns (not significant), comparing FRDA to control)

Both experimentally in animal models [18] and human disease [22] evidence suggests that inflammatory cues promote BMDCs to fuse with Purkinje cells to form bi-nucleated Purkinje cell heterokaryons. In control sections immuno-labelled for the macrophage/microglial markers HLA-DP, DQ, DR, few positive cells were detected throughout the cerebellar folia (Fig. 6a and b). In FRDA cases, the overall frequency of HLA-DP, DQ, DR-positive microglia was highly variable between patients and no significant differences in microglial numbers were observed, in both the white matter and grey matter regions, between FRDA and control cases (Fig. 6a and b).

Using correlative analysis to investigate the relationship between microglial numbers and the incidence of bi-nucleated Purkinje cells, we found a significant positive correlations in the grey matter between the levels of HLA-DP, DQ, DR-positive cells and both the frequency of total bi-nucleate Purkinje cells and bi-nucleate Purkinje cells containing one large and a second smaller nucleus (*p* <0.05) (Fig. 6c). No correlation between HLA-DP, DQ, DR-positive cells and the frequency of bi-nucleate Purkinje cells containing two large nuclei was observed.

## Discussion

Here we present a pathological study showing novel and fundamental insights into Purkinje cell injury, remodelling and spontaneous repair in FRDA. We provide evidence of significant injury to the Purkinje cell axonal compartment with relative preservation of the perikaryon and its extensive dendritic arborisation. Axonal remodelling of Purkinje cells was clearly apparent. We have also shown a considerable increase in the frequency of bi-nucleate Purkinje cell heterokaryon formation in FRDA cases. This is the first time in humans that Purkinje cell fusion and heterokaryon formation in the brain has been reported in a genetic condition. We also provide evidence that heterokaryon formation is influenced by underlying levels of cerebellar inflammation associated with the disease. Our results together demonstrate the Purkinje cell’s unique plasticity and regenerative potential in response to damage.

Atrophy of the dentate nucleus, which receives inhibitory GABA-ergic inputs from Purkinje cells originating within the cerebellar hemispheres, is the most significant change seen within the central nervous system of patients with FRDA [26–28, 30, 31, 49]. In FRDA cases, where the dentate nucleus was visible, we observed archetypal neuronal loss accompanied by sites of grumose degeneration. This pathological transformation labelled positively for Calbindin-D_28k_ demonstrating that abnormal axon terminals were of Purkinje cell origin. The resulting synaptic detachment of Purkinje cell axon terminals from degenerating neurons within the dentate nucleus would likely result in a retrograde change and a lack of synaptic contact with the efferent target may lead to trans-neuronal degeneration of the Purkinje cell. However, contrary to pathological nomenclature, recent reports suggest grumose degeneration may actually be a beneficial phenomenon in which Purkinje cells escape retrograde atrophy by producing new axonal sprouts that establish contact with small surviving neurons where they form reparative grumose clusters [30].

Indeed, there were marked changes to the Purkinje cell axonal compartment, with abnormal axonal phosphorylation and abundant axonal thickening/formation of axonal torpedoes within the granular cell layer. It is thought in disease states that abnormal accumulation of the axonal structural components leads to morphological changes within the axon, and depending on the underlying disease mechanism, the accumulated material can differ [35, 57]. The axon torpedo (a fusiform swelling of the axon) is generally characterised by a central accumulation of disoriented neurofilaments, which displace the mitochondria and endoplasmic reticular elements to the periphery [37]. Neurofilament structures are essential for axonal maintenance and both anterograde and retrograde axonal transport. It is conceivable that loss of dentate nucleus neurons results in localised failures of axonal transport causing the build-up of transported proteins and axonal swelling [9]. We found frequent phosphorylated neurofilament accumulation within both hypertrophic axons and axon torpedoes, and to a lesser extent within the perikarya. Neurofilament phosphorylation and rates of axonal transport inversely correlate, this again signifying an impaired axonal transport within these areas [19, 41]. It is not known pathologically exactly how these neurofilaments become aberrantly phosphorylated, however accumulation of phosphorylation is a hallmark of several neurodegenerative conditions, including ALS [40], Alzheimer’s disease [50] and multiple sclerosis [16, 44]. Elevated phosphorylated neurofilaments detected in the CSF can be used as early protein biomarkers in disease and even correlate with clinical disability [42], together highlighting the critical role neurofilament changes have on both axonal damage and neurodegenerative processes.

The typical response of most neurons to axonal injury is a cell body reaction involving a complex sequence of molecular and genetic changes that initially sustain compensatory and regenerative responses, but eventually become regressive leading to atrophy or cell death [45]. However, cerebellar atrophy is not a major pathological characteristic associated with FRDA. Purkinje cell survival was not significantly reduced in FRDA cases and morphological analysis showed no signs of pruning to the Purkinje cell dendritic arbour. In FRDA cases the global perikaryon size was also unchanged, however in a sub-group of Purkinje cells with an associated axon torpedo, a significant reduction in perikaryon size was evident. Elevated levels of Purkinje cell perikarya with abnormal deflated/shrunken morphologies and/or SMI-34 positivity were observed, which may indicate early changes leading to cell death or possible compensatory structural modifications due to the close proximity of the axonal obstruction. Purkinje cells have an inherent and atypical resistance to stress within the CNS and often survive despite injury to their axon [12]. Purkinje cells in FRDA cases showed remarkable morphological plasticity in response to injury in the form of axonal branching, arciform fibres, hypertrophic recurrent collaterals and axonal sprouting. These changes were abundant and are thought to allow the Purkinje cell to draw retrograde trophic support by way of either reorganisation of the intra-cortical plexus or establishing new connections with other Purkinje cells or neurons in the surrounding granular layer, thus dampening down a cell body response to injury [43].

Remodelling of the Purkinje cell axon can be induced by extrinsic inhibitory cues such as myelin-associated molecules. For example, blocking of myelin-associated neurite growth inhibitory protein (Nogo-A) binding to it receptor complex situated on the axonal membrane, can induce prolific spouting in Purkinje cells from both the corticofugal axon and the intra-cortical plexus [5, 14]. Nogo proteins in the adult CNS are now known to be negative regulators of neurite growth, leading to stabilization of the CNS wiring in the healthy brain at the expense of extensive plastic rearrangements and regeneration after injury [47]. In relation, we observed significant amounts of both myelin fragmentation and loss localised to both hypertrophic axons and axonal torpedoes within the granular layer. It could therefore be hypothesized that an increase in axon calibre disrupts myelin/axon interactions and is, at least in part, responsible for initiating a cell survival response through promoting axonal sprouting and remodelling.

Of major relevance to ataxia is the potentially neuro-regenerative process of BMDCs migrating into the CNS and fusing with, in particular, Purkinje cells within the cerebellum. A process in which the two distinct cells fuse together to become a single cell, resulting in transfer and amalgamation of nuclear material, to form bi-nucleate or mono***-***nucleate cells. A key observation in this study was evidence of Purkinje cell fusion in diseased FRDA brains through detecting bi-nucleate Purkinje cell heterokaryons; the frequency of these cells in FRDA being significantly higher than that previously reported in control cases [22]. The phenomenon of BMDCs fusing with Purkinje cells has been observed in a variety of experimental models of cerebellar disease [2, 3, 8, 10, 11] and the presence of cell fusion, shown also by detection of increased bi-nucleated neurons, has been described in a variety of human CNS diseases, including multiple sclerosis [22], Alzheimer’s disease [58] and *Spino-olivo-ponto-*cerebello*-nigral atrophy* [20].

Purkinje cells are generated only during early brain development and in contrast to other neurons there is no evidence of their generation after birth [53]. Under normal physiological conditions Purkinje cell fusion seems to occur at very low levels [53, 54]. In FRDA cases, we found no correlation between patient age and the frequency of fusion, though experimentally, the incidence of cell fusion in the brain is enhanced with age, radiation exposure, inflammation, chemotherapeutic drugs and even selective damage to the neurons themselves [13, 18, 21, 36, 54, 55]. This raises the possibility that fusion represents a rescue process, both in FRDA and other neurological conditions, by which BMDCs migrate into the CNS and donate genetic material to injured highly complex cell types that otherwise cannot be replaced in adults through classical modes of trans-differentiation [4, 48]. Indeed, in organs other than the brain, fusion clearly represents a crucial process by which degenerating cells with genetic damage can be rescued, promoting their survival [32, 52, 56]. Using genetic models of Purkinje cell degeneration, researchers have explored the potential of using BMDCs to protect Purkinje cells. These studies have provided compelling evidence for BM cell-mediated neuroprotective mechanisms, with a neurodegenerative microenvironment appearing to augment Purkinje cell fusion [2, 10, 11]. Further studies also showed that transplantation of enhanced green fluorescent protein (EGFP)-expressing BMDCs directly into the cerebellum alleviates Purkinje cell degeneration in murine Niemann-Pick Type C1 disease. Moreover, fusion between these BMDCs and existing Purkinje cells led to the formation of electrically active neurons with functional synaptic formation within a neurodegenerative cerebellar environment [3].

Little is known concerning the regulatory mechanisms behind controlling Purkinje cell fusion in response to injury; however, inflammatory cues are known to play a physiologically significant role. Fusion between Purkinje cells and BMDCs occurs ordinarily at low levels, but the incidence of fusion is substantially increased by chronic inflammation [18, 21]. The mechanisms by which inflammation enhances fusion are unclear, but the combination of TNF-alpha and IFN-gamma particularly enhance the process *in vitro* [21]. We found the degree of neuro-inflammation, characterised by increased microglial cell numbers, to be highly variable within FRDA cases and not significantly elevated compared to controls. In contrast to FRDA-affected cardiac tissue [29], the cerebellum does not characteristically show a significant inflammatory component, although microglial changes in ferritin immunoreactivity, morphology and frequency have previously been reported [28, 31]. Interestingly, in the FRDA cases used for our study, we did find a significant correlation between microglial numbers within cerebellar grey matter and the formation of bi-nucleate Purkinje cell heterokaryons. More specifically, microglial cell numbers correlated with numbers of bi-nucleate Purkinje cells harbouring both a large Purkinje cell-like nucleus with disperse chromatin and a second smaller nucleus with compact chromatin; a nuclear phenotype signifying a recent fusion event; the donated nucleus yet to be reprogrammed to finally assume the morphology of a Purkinje cell nucleus. Purkinje cells are classically mono-nucleate cells [38]. It is hypothesised that once fusion has occurred the small dense donor nuclei is progressively reprogrammed, causing it to become, over time, less dense and compact, and finally to assume the morphology of the Purkinje nucleus [54]. Hence, the nuclear phenotype of fused cells can be an indication of heterokaryon maturity. Due to the chronic nature of the disease, it is not surprising that the large majority of bi-nucleate cells in FRDA cases contained two large Purkinje cell-like nuclei. However, finding an association between existing grey matter microglial levels and recent fusion events provides compelling evidence that local inflammatory cues, albeit at the end stages of the disease, can promote Purkinje cell fusion in humans.

## Conclusion

Whether the observed changes to Purkinje cells in our FRDA cohort are solely a response to depleted cellular frataxin levels and/or loss of the dentate nucleus; or alternatively, at least in part, a result of secondary damage caused by hypoxic-ischemic injury (due to either cardiomyopathy or pulmonary complications as reported in our FRDA cases) cannot be fully determined. As yet, the precise functional significance of Purkinje cell plasticity in response to stress/injury is also unknown. Nevertheless, it is likely to be closely linked to the central role Purkinje cells play in maintaining cerebellar function. It is tempting to speculate the reasons for such phenomena; survival of Purkinje cells in FRDA may be functionally irrelevant as neuronal loss within the dentate nucleus blocks corticonuclear transmission. However, these fairly unique changes in response to axonal injury may allow the Purkinje cell to convey its output information through nearby intact neurons, or be a neuroprotective process to prevent a further cascade of disabling neurodegenerative changes, as would likely result from a loss of Purkinje cells, within the cerebellar milieu. More research into these mechanisms are warranted and elucidating the biological pathways behind both neuronal plasticity and cell fusion in disease could have significant clinical implications in manipulating neuronal repair in response to injury.
