# Erratum to: ‘Purkinje cell injury, structural plasticity and fusion in patients with Friedreich’s ataxia’

**DOI:** 10.1186/s40478-016-0332-5

**Published:** 2016-06-30

**Authors:** Kevin C. Kemp, Amelia J. Cook, Juliana Redondo, Kathreena M. Kurian, Neil J. Scolding, Alastair Wilkins

**Affiliations:** Multiple Sclerosis and Stem Cell Group, School of Clinical Sciences, University of Bristol, Bristol, BS10 5NB UK; Brain Tumour research group, School of Clinical Sciences, University of Bristol, Bristol, BS10 5NB UK

## Erratum

After publication of this study [1], we discovered that the unit of measurement used to quantify changes in axonal morphology had been incorrectly labelled. Both the methods section ‘Purkinje cell axonal morphology’ and ‘Table 2 legend’ should read:- All axonal changes are expressed as either frequency per standardized unit length of the Purkinje cell layer (1 centimetre) or frequency per Purkinje cell.

## Competing interests

The authors declare that they have no competing interests.

## References

[1] Kemp KC, Cook AJ, Redondo J, Kurian KM, Scolding NJ, Wilkins A (2016). Purkinje cell injury, structural plasticity and fusion in patients with Friedreich’s ataxia. Acta Neuropathol Commun.

